# Incorporation of Tannic Acid in Food-Grade Guar Gum Fibrous Mats by Electrospinning Technique

**DOI:** 10.3390/polym11010141

**Published:** 2019-01-15

**Authors:** Weiqiao Yang, Min Zhang, Xihong Li, Jianan Jiang, Ana M.M. Sousa, Qiang Zhao, Sherri Pontious, LinShu Liu

**Affiliations:** 1 State Key Laboratory of Food Nutrition and Safety, School of Food Engineering and Biotechnology, Tianjin University of Science and Technology, Tianjin 300457, China; yangweiqiaospring@126.com (W.Y.); zm0102@tust.edu.cn.com (M.Z.); jiangjianan111@126.com (J.J.); 2 Tianjin Jiesheng Donghui Fresh-keeping Technology Co., Ltd, Tianjin 300403, China; 3 Dairy and Functional Foods Research Unit, United States Department of Agriculture, Agricultural Research Service, Eastern Regional Research Center, 600 East Mermaid Lane, Wyndmoor, PA 19038, USA; ana.sousamm@gmail.com (A.M.M.S.); LinShu.Liu@ARS.USDA.GOV (L.S.L.); 4 State Key Laboratory of Medicinal Chemical Biology, Key Laboratory of Bioactive Materials, Ministry of Education, College of Life Sciences, Nankai University, Tianjin 300071, China; qiangzhao@nankai.edu.cn; 5 Department of Microbiology and Immunology, Temple University School of Medicine, Philadelphia, PA 19140, USA; sherriannpontious@gmail.com

**Keywords:** electrospinning, guar gum, tannic acid, antioxidant, nanofiber

## Abstract

The use of polysaccharides to produce functional micro- or nanoscale fibrous mats has attracted growing interest for their food-grade applications. In this study, the characterization and electro-spinnability of guar gum (GG) solutions loaded with tannic acid (TA) was demonstrated. Food-grade antioxidant materials were successfully produced by electrospinning while incorporating different loads of TA into GG fibers. Bead-free GG-TA fibers could be fabricated from GG solution (2 wt %) with 10 wt % TA. Increasing the amount of TA led to fibers with defects and larger diameter sizes. Fourier Transformed Infrared Spectroscopy and X-ray Diffraction of neat GG and TA loaded GG fibrous mats suggested that inclusion of TA interrupted the hydrogen bonding and that a higher density of the ordered junction zones formed with the increased TA. The high TA incorporation efficiency and retained antioxidant activity of the fibrous mats afford a potential application in active edible film or drug delivery system.

## 1. Introduction

The interrelationship between structure and physicochemical characteristics for delivery and encapsulation materials requires the design to employ three key characteristics: (1) matrices with high porous structure and huge surface area–volume ratio to permit medium, relatively free exchange between cellular constructs, due to low permeability resistance [1,2]; (2) morphology-controlled process and toxic reagent-free methods, due to potential human and environmental safety concerns; and (3) greater incorporation of instructive molecules such as food additives or drugs to offer significant opportunities for enhancing the functionality [3]. 

Among various methods, the electrospinning technique has attracted growing interest to produce such materials and proven to be a relatively efficient and versatile nanotechnology to develop continuous fibers with diameters down to nano- to micrometer dimensions [4,5,6], which afford inherently high surface area–volume ratios and have excellent mass transfer properties [7]. Functional or unstable agents can be encapsulated with these fibers by mixing therapeutic additives with biopolymers when spinning, with improved bioavailability and controlled release [8,9,10]. In terms of functional delivery system, the microstructure has shown an important impact on digestion and absorption of bioactive compounds in the digestive tract [11,12]. However, most of the reported nanofibers are fabricated from synthetic polymers, in which organic solvents were commonly used to dissolve polymers, it is still challenging to produce 100% food-grade nanofibers using natural materials such as polysaccharides.

Guar gum (GG), which is one type of galactomannan acquired from the seeds of *Cyamopsis psoraloides*, is a naturally occurring hydrocolloid polysaccharides [13,14]. Due to its natural nontoxic, biodegradable, and cost-effective properties, GG has been considered a substitute for starch in food industry because this dietary polysaccharide is admittedly resistant to human digestion and absorption, with cholesterol and glucose lowering effects [15,16]. It also has been widely employed as food additives to affect the physicochemical properties of various food products such as ice cream, bakery products, beverages, etc. [17]. Oral administration using GG as the delivery system for drugs show that GG could release active agent in the colon and delay their release in target site under conditions mimicking mouth to gastrointestinal tract [18]. Hence, it occupies superior position in the food industry as a functional ingredient owing to its dietary fiber characteristic [19,20,21], as well as in the pharmaceutical field for drug delivery systems [22,23], which makes it an ideal carrier candidate to encapsulate a variety of bioactive food ingredients. The potential for electrospun GG has not yet been fully realized due to its high viscosity at a comparatively low content. The poor spinnability of GG is owing to it supra-molecular aggregate structures consisting of galactomannan molecules that interact with each other through hydrogen bonding [24,25]. Some researchers have used gums to generate nanofibrous polysaccharides-based materials for biomedical applications, but synthetic biopolymers (e.g., polyvinyl alcohol) are still needed as a carrier to overcome the poor fiber forming ability [26,27]. Although purification and filtration processes are used to improve the morphology [25], it is still hard to develop the continuous uniform nanostructure of GG fiber, much less use it to deliver functional additives. 

Tannic acid (TA) is a natural phenolic compound that is ubiquitously distributed in an abundance of fruits and plants. Because of its ability to inhibit the hydroxyl radical formation by providing an electron or hydrogen atom, numerous studies have shown that TA exhibits excellent antioxidant, anticarcinogenic, and antimicrobial properties [28,29,30]. These biological advantages make TA a promising option for the fabrication of functional additives [30,31]. The plant-derived components (GG and TA) are both recognized as safe by FDA and even edible. The use of natural polysaccharides to develop active materials has a wide potential application in drug delivery systems [7], functional foods [32] and active edible packaging [33]. 

In this study, we examined the feasibility of incorporation of TA to develop electrospun GG fibrous mats with antioxidant activity for the first time. Prior to electrospinning, GG molecular entanglement in fiber formation was investigated. Next, 100% food-grade antioxidant materials were successfully produced by electrospinning, while incorporating different loads of TA into GG fibers. For the resultant fibrous mats, incorporation of TA in the fiber formation and physical characteristics were examined, including morphology, size, crystallinity, thermal stability, antioxidant activity, etc. The use of an efficient and organic solvent-free electrospinning method to fabricate edible functional GG fibrous mats demonstrates the potential for green electrospinning and its food-grade applications.

## 2. Materials and Methods

### 2.1. Materials

Tannic acid (TA, C_76_H_52_O_46_) and commercial guar gum (GG, molecular weight of Mw = 3.896 × 10^6^ g/mol) were obtained from Sigma-Aldrich (Saint Louis, MO, USA). Ethanol (ACS grade) was obtained from Tianjin Sixth Chemical LTD (Tianjin, China). 1,10-diphenyl-2-picrylhydrazyl (DPPH) was purchased from TCI Shanghai (Shanghai, China). All aqueous solutions were prepared with MilliQ water (resistivity of 18.2 MΩ) from a Barnstead E-pure water system (Dubuque, IA, USA).

### 2.2. Preparation of GG and GG-TA Spinning Solution

#### 2.2.1. Purification of GG

Commercial GG needed to be purified before use to remove impurities existing in the powder. The purification of GG was according to Lubambo’s report by ethanol abstraction method with some modification [25]. In brief, the GG was slowly dispersed into a vigorous vortex of Millipore water (~1.3 wt %) at room temperature and continuously stirred (700 rpm/min) overnight using a magnetic stirrer until complete polymer dissolution was achieved. The mixture was then transferred into the centrifuge tubes and centrifuged at 40 °C at 8000 rpm/min for 40 min. A 500 mL dripping funnel was used to contain the supernatant and control its slow precipitation into ethanol at a 1:2 volume ratio. This process was under continuous stirring until the completion of the precipitation. The obtained white particles were collected and washed with ethanol three times followed by suction filtration, and then dried for 24 h at room temperature in a vacuum oven. The resulting pure GG was ground into powder for further use.

#### 2.2.2. Preparation of GG and GG-TA Spinning Solutions

A series of GG solutions with different concentrations (0.6–2.2 wt %) were prepared by dispersing a certain amount of the purified GG powder into water at 50 °C. To completely dissolve the GG, the mixtures were incubated overnight at the same temperature with gentle magnetic stirring (700 rpm/min). To study the effects of incorporation of TA on fiber formation, four solutions varying by 5% of TA loaded GG spinning solution were prepared prior to electrospinning. These solutions were designated as GG-TA 5%, GG-TA 10%, GG-TA 15%, and GG-TA 20%, respectively. They were prepared by mixing precise amounts of TA with concentrations ranging from 5 to 20 wt % (based on the weight of dry GG) with adequate GG spinning solutions. The mixtures were constantly stirred (1000 rpm/min) at 50 °C for 3 h using a magnetic stirrer until homogeneous mixtures were obtained. 

### 2.3. Viscosity Measurements

Viscous behavior was analyzed using the shear rheology test and performed through a Kinexus Rheometer (Malvern Instruments, Worcestershire, UK) by using an attached cone-plate geometry (diameter 40 cm, 2° θ, truncation gap 54 μm). The prepared GG solutions ranging from 0.6 to 2.2 wt % were carefully loaded on the Peltier stage (preheated to 50 °C) and subsequently covered with paraffin oil to prevent the solution from drying. The apparent viscosity of the sample, responding to steady shear flow was analyzed with increasing shear rate from 0.001 to 1000 s^−1^.

The Cross–Williamson model was used to calculate the zero-shear viscosity (η_0_) from the flow curves as the following equation according to previous report [34]:

(1) $$\frac{\eta_{\mathrm{app}}}{\eta_{0}}=\frac{1}{1+k{\dot{\gamma }}^{\left(1-\nu \right)}}$$

where η_app_ represents the apparent viscosity, η_0_ represents the zero-shear viscosity, *k* is the time constant, γ represents the shear rate, and n indicates the flow index.

By using the η_0_, the specific viscosity (η_sp_) was obtained by the following formula [35,36]:

(2) $$\eta_{\mathrm{sp}}=\frac{\eta_{0}-\eta_{s}}{\eta_{s}}$$

where η_s_ is the solvent viscosity.

### 2.4. Electrospinning

The electrospinning process was carried out on a YF SP-T unit (Yunfan Technologies Co., Limited, Shenzhen, China). Approximately 3 mL of the composite solution were loaded onto a 5 mL plastic syringe, attached with a stainless-steel needle placed in the spinneret. The conducting needle had an outer diameter of 0.7 mm and inner diameter of 0.40 mm. Through a series of pretests under various process parameters, the optimum electrospinning condition in this work was fixed at: applied voltage, 18 kV; distance tip-to-collector, 10 cm; feed rate 0.5 mL/h; and drum rotating speed, 40 rpm/min. For all cases, the electrospinning temperature was set at 50 °C to prevent the formation of gel when the solutions were ejected from the tip of spinneret. After continuously electrospinning for 6 h, fibrous mats were collected on the aluminum foil that covered the surface of the drum. The obtained mats were stored at controlled relative humidity (53%) for 24 h before being tested.

### 2.5. Characterization of Nanofibers

#### 2.5.1. Scanning Electron Microscopy (SEM)

Prior to imaging, samples were fixed on the specimen stubs by double sided tape and then coated with gold sputter. The morphology of the nanofibers was characterized through a 0182-S Phenom-World Scanning Electron Microscope (SEM, Thermo Fisher Scientific, Pittsburgh, PA, USA) following our previously reported procedure [31,37]. The images were acquired with the working condition of the instrument set at: accelerating voltages, 10 kV; high-vacuum/secondary electron imaging mode; and working distance, 10.1–11.4 mm. The average fiber diameters were analyzed using ImageJ Software v1.45 (NIH, Bethesda, MD, USA). The mean values of the fiber diameters, along with standard deviations of distributions, were determined after measuring 200 fiber diameters at random.

#### 2.5.2. Incorporation Efficiency of TA

The TA content in the solution was analyzed using the spectrophotometric method according to our previous report [31,37], and the incorporation efficiency (IE) of TA in the fibers was obtained based on the following equation:

(3) $$\mathrm{IE}\%=\frac{\mathrm{Actual}\;\mathrm{TA}\;\mathrm{concentration}}{\mathrm{Theoretical}\;\mathrm{TA}\;\mathrm{concentration}}\times 100\%$$

#### 2.5.3. Fourier Transformed Infrared Spectroscopy Analysis

The infrared spectra of the GG-TA fibrous mats were examined using a Nicolet iS50 Fourier transform infrared (FT-IR) spectroscopy (Thermo Scientific, Pittsburgh, PA, USA) with a diamond Attenuated Total Reflectance (ATR) system attached. The fibrous mats were scanned at 0–4000 cm^−1^ wavelength and OMNIC (spectral signal processing software) was used to obtain the transmittance curve of the sample at different wavelengths.

#### 2.5.4. X-Ray Diffraction (XRD) Measurements

The XRD patterns were recorded using a Philips Analyzer PW 1830 X-ray VB (1.54 A, Philips, Amedo, the Netherlands) with Cu-Kα radiation. Scans were obtained in the 2*θ* range with a step size of 0.01° per 1 s. The scanning rate was set at 2°/min.

#### 2.5.5. Thermal Properties

Differential scanning calorimetry (DSC) was carried out on the pure GG powder and the as-spun electrospun fibrous mats by a DSC 204 F1 system (Netzsch, Germany). The thermal analysis for all samples (2.5 mg) were performed at a 10 °C/min heating rate with increasing temperature from 50 to 250 °C in nitrogen.

#### 2.5.6. Antioxidant Activity

The antioxidant activity of the fibrous membranes was measured by means of 1,10-diphenyl-2-picrylhydrazyl (DPPH), as reported by Neo et al. [25]. Briefly, a 10 mg fiber membrane was accurately submerged in a tube containing 10 mL of 80% aqueous ethanol solution. After mixing completely in a vortex, 100 μL of each solution was used to react with 3 mL DPPH solution (0.1 mM) for 30 min in the dark. The absorbance was obtained via a UV spectrophotometer at an absorbance of 516 nm. The antioxidant activity of the fibrous membranes was expressed as a percentage of DPPH radical inhibition (%AA) according to the following equation:

(4) $$\%\;\mathrm{AA}=\frac{A\;\mathrm{control}\;-A\;\mathrm{sample}}{A\;\mathrm{control}}\times 100\%$$

where A _control_ and A _sample_ represent blank absorbance and the values of the DPPH solution combined with the sample for each reaction, respectively. Experiments were tested in triplicate.

### 2.6. Statistical Analysis

The results were acquired using SPSS Statistics 22 software (IBM, Amun, NY, USA). One-way ANOVA with Tukey’s multiple comparisons test (*p* < 0.05) was carried out to analyze the significance of fiber diameter, incorporation efficiency of TA, and antioxidant activity. 

## 3. Results and Discussion

### 3.1. Viscosity and Spinnability of GG Solutions

TA is a non-polymeric functional molecule. According to our previous experiment [31], TA did not have any fiber-forming ability within the required concentration range because of its relatively low viscosity. In this work, natural polymer GG was used as the structure-forming matrix to avoid the breakup of the fluid jet into droplets during electrospinning. Despite previous report that GG solutions have fiber-forming ability [25], the scope to form the fibers had not yet been investigated. Therefore, the relationship between the rheology and the spinnability of GG was preferentially explored to find optimal concentration to load TA under stable electrospinning process.

**Figure 1:**
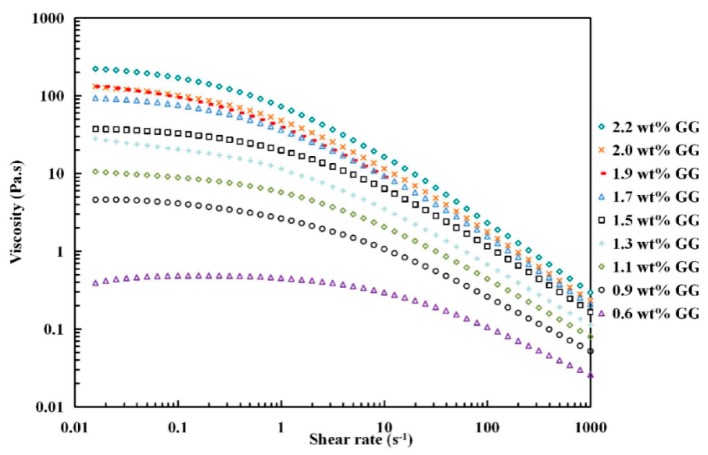
Apparent viscosity of GG solutions of different concentrations as a function of shear rate.

As depicted in Figure 1, the apparent viscosity of GG solutions at each shear rate strongly depended on its concentration, in general increasing as concentration increased. The flow curves were carried out at 50 °C. In all cases, GG solutions exhibited typical non-Newtonian (pseudoplastic) behavior with increasing shear rate (> s^−1^), in which the shear viscosity reduced with shear rate and became noticeable with increasing GG concentration, displaying shear thinning property. Similar results were reported by Martín-Alfonso [38] and Torres et al. [34]. Shear thinning is often explained to be the result of breakdown of interchain interactions in the polymer network, i.e. random movement of polymer chains become more progressively limited as the concentration increases and consequently the rate to reform new entanglements is lower than those unwinding under high shear stress, resulting in a decrease interaction in the polymer network as the concentration increases [39,40]. 

**Figure 2:**
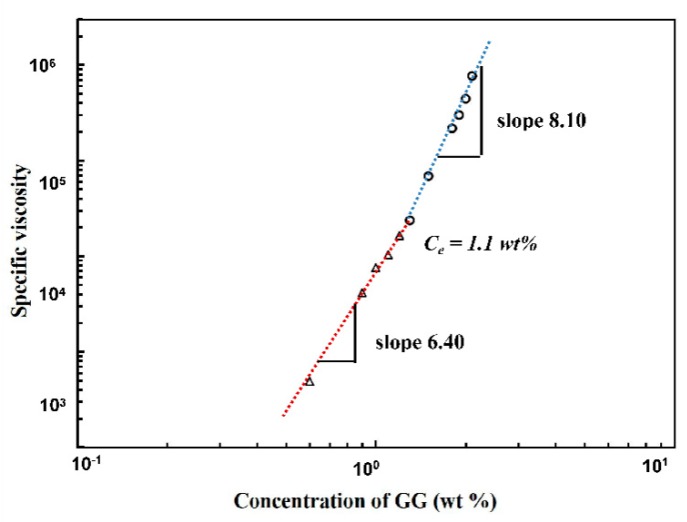
Plot of specific viscosity versus aqueous GG concentration.

Flow curves of GG solutions mentioned above showed a satisfactory fitting with the Cross–Williamson model (R^2^ > 0.99) in the shear rate range and the resultant zero-shear viscosities η_0_ were used to further obtain specific viscosity values. By using the specific viscosity, chain entanglement concentration Ce was calculated from the plots of specific viscosity η_sp_ data versus the GG concentration in double logarithmic coordinate for these polysaccharide solutions [35,41,42]. In electrospinning process, Ce is the minimum concentration before attaining stable electrospinning, with possible fabrication of uniform bead-free nanofibers [43,44]. As shown in Figure 2, the Ce was determined to be ~1.1 wt % for the GG solution from the crossover of two different linear intervals. At low concentrations, specific viscosity η_sp_ ~ c ^6.4^ was observed, indicating GG solutions in this range were in semi-dilute unentangled state, in which the chain overlap between individual molecules was insufficient to form any notable degree of entanglement. Above Ce, a notable increase in gradient was obtained and reached a constant slope of 8.10, suggesting the entrance of the semi-dilute entangled regime. The Ce detected in this study was slightly higher than Torres’s report, where GG concentrations between 5 and 10 g/L were suggested to be in the entangled regime when the test was carried out at 20 °C. The variation of Ce might due to the difference of sample temperature under test.

### 3.2. Morphology of Electrospun GG Fibers

**Figure 3:**
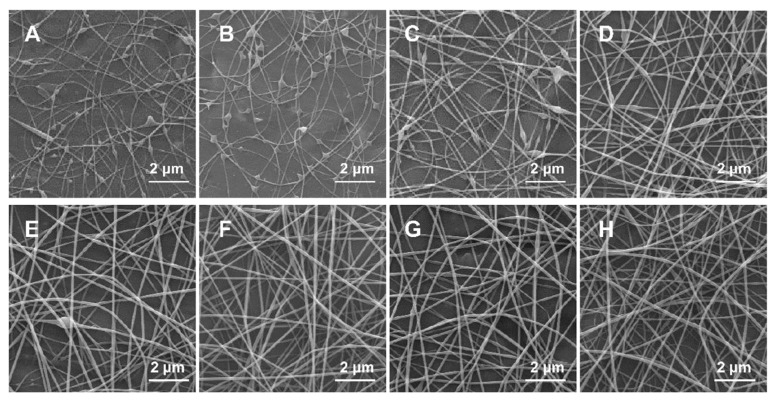
SEM images of electrospun fibers obtained from GG solutions with concentration of: (**A**) 1.1 wt %; (**B**) 1.3 wt %; (**C**) 1.5 wt %; (**D**) 1.7 wt %; (**E**) 1.9 wt %; (**F**) 2.0 wt %; (**G**) 2.1 wt %; and (**H**) 2.2 wt %. Magnification is 10,000×.

A series of GG solutions above the critical entanglement concentration (1.1 wt % GG) were electrospun. During the spinning, a continuous jet with stable whipping movements could eject from the spinneret at the concentration range between 1.1 and 2.2 wt %, allowing orderly deposit of fibers on the collector. However, it was difficult to continuously spin GG solutions when concentration was raised above 2.5 wt % since the solutions became too viscous, in which gel formed on the tip of needle and electro-spraying constantly occurred. The representative microscopic images of the collected fibers were examined by SEM, as shown in Figure 3. At entanglement concentration (1.1 wt %), the resulting electrospun fibers consisted of more beads with limited incipient fibers (Figure 3A). The formation of GG fibers became obvious when the concentration roswas raised to 1.5 wt %, but most fibers exhibited a beaded-on-string morphology (Figure 3C). The transition from beaded-on-string structure to defect-free fibers occurred at 2 wt % of GG solution, which was the minimum threshold that yielded smooth and uniform microstructure (Figure 3F), indicating molecules of GG exhibit enough intermolecular force to stable extensional deformation caused by the applied electric filed. Experimentally, the concentrations to obtain defect-free or beadless fibers here were found at 1.8–2.3 times Ce, which was close to that of other biopolymer solutions in previous reports [6,35,45].

The homogeneous morphology of GG obtained from purified GG solution had different results in some aspects from Lubambo’s results [25]. In particular, the uniform fibers could be obtained in a more simplified process without using any filtration procedure. This disparity might result from the preparation method, detailed purification process of the solution and parameters of electrospinning. 

### 3.3. Effect of Incorporating TA on the Morphology of GG-TA Nanofibers

**Figure 4:**
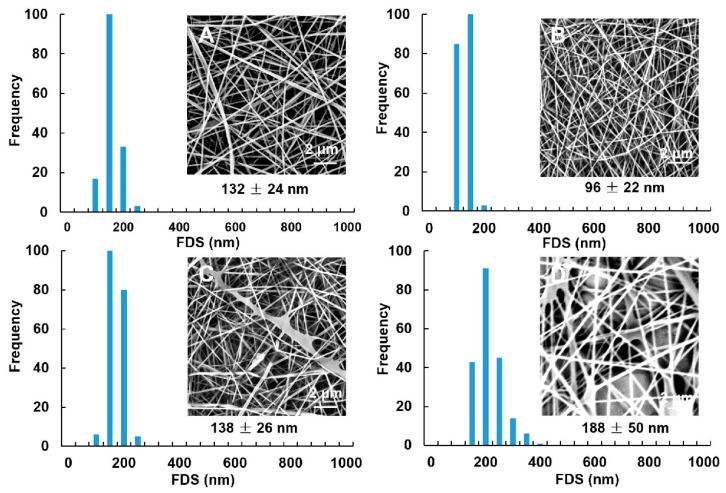
SEM images and fiber diameter size (FDS) of electrospun fibers obtained from GG solutions at 2 wt % concentration loaded with: 5 wt % TA (**A**); 10 wt % TA (**B**); 15 wt % TA (**C**); and 20 wt % TA (**D**). Magnification is 10,000×.

In blended GG-TA solutions, 2 wt % of GG solution was chosen as the carrier to incorporate different loading amount of natural antioxidant TA. Fiber forming ability of GG-TA solutions was explored as a function of TA concentration under the same spinning parameters with GG. As depicted in Figure 4, it was determined that inclusion of TA has a dramatic effect on the morphology of the fibers as well as the fiber diameter distribution. The average diameter size ranged from 96 to 188 nm and followed non-normal distributions (*p* < 0.05). The incorporation of TA did not produce visible defects in the GG fibers when the TA increased from 5 to 10 wt % (compared Figure 4A,B), while obvious aggregation formed as the concentration of TA increased to 15 wt % (Figure 4C). In addition, the average diameter increased from 138 to 188 nm as the concentration of TA increased from 15 to 20 wt % (*p* < 0.05; Figure 4D) in comparison to 10 wt % (Figure 4B). Stable electrospinning could be carried out for several hours to finally obtain fibrous mats. It is worth noting that the formation of nanofibers in this system was manufactured using water as the solvent without using any synthetic chemicals. These results are encouraging findings for green electrospinning and its potential food-grade applications. 

### 3.4. Loading Efficiency of TA

Our pretests using the spectrophotometer have shown that both the TA standard and the fibrous mats loaded with TA have their maximum absorbance observed at 275 nm [31], while neat GG fibrous mats showed no absorption in this range. The loading efficiency of TA was calculated from the linear regression equation of the standard curve, as listed in Table 1. The results confirm the existence of TA in fibrous mats and show that all of the GG-TA fibrous mats had high loading efficiency, close to 100%. Another research group obtained similar results, finding that the loading efficiency for gallic acid varied from 99% to 105% in a zein-gallic acid electrospun fibers system [11]. These results suggest that the electrospinning technique is feasible for the given delivery system and can efficiently incorporate instructive molecules to produce functional micro- to nanoscale fibrous mats. Encapsulation of this food-grade antioxidant in the nano-scale delivery systems may exhibit a unique structure compared with macro antioxidants, potentially applying in prolong shelf life of foods as edible packaging or functional foods.

### 3.5. Interactions between TA and GG in GG-TA Fibrous Mats

**Figure 5:**
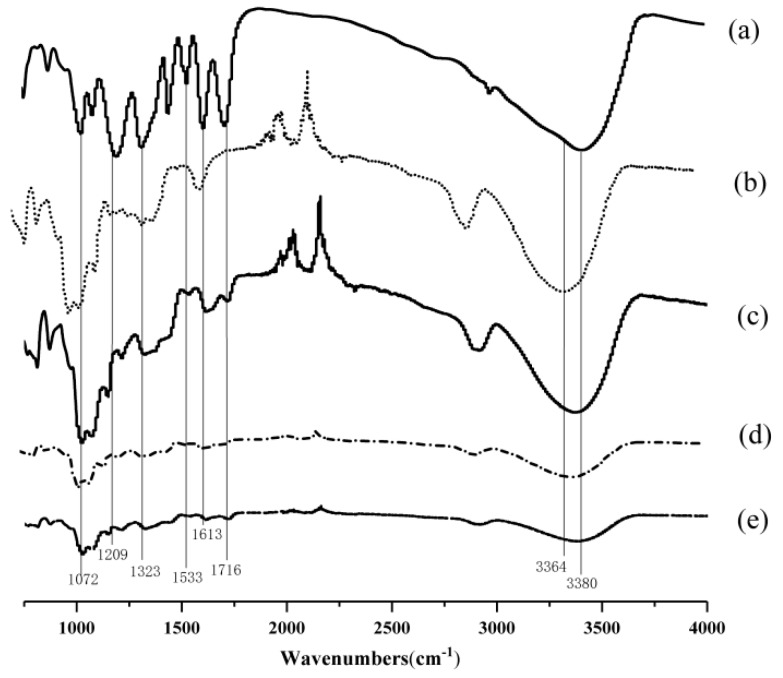
FT-IR spectra of the: TA powder (a); neat GG (b); and of GG fibrous mats loaded with: 10% TA (c); 15% TA (d); and 20% TA (e).

To assign intermolecular interactions between TA and GG, as-spun GG fibrous mats with and without incorporating TA were characterized using FT-IR spectra. In Figure 5, Curves a and b refer to the spectra of TA powders and neat GG fibrous mats, respectively, while Curves c–e represent the spectra of GG fibrous mats incorporated with 10, 15, and 20 wt % TA, respectively. The characteristic absorption peak of TA was identified using FT-IR examination (Figure 5, Curves c–e vs. Curve a) through the presence of C=O stretching vibration of ester group (1716 cm^−1^), aromatic –C=C– stretching vibration (1613 and 1533 cm^−1^), and O−H stretching of alkylphenol (1323 cm^−1^), indicating the presence of TA loaded on electrospun GG fibrous mats.

The increase in TA led to a marked difference in the FT-IR spectra of the GG-TA fibrous mats. The broad peaks that occurred at 3364 and 3380 cm^−1^ were related to the stretching mode of interchain interaction via hydrogen bonding, and became weaker when TA increased from 10 to 20 wt % (Figure 5c–d). Additionally, the peaks at 1209 and 1323 cm^−1^, due to O−H vibration from phenolic hydroxyl of TA, were less discernible in the fibers incorporated with TA when the TA increased to 15 wt % than with 10 wt % (Figure 5, Curve d vs. Curve c) and neat GG fibrous mats (Figure 5, Curve b). These peaks did not change noticeably with 20 wt % of TA loading. Similarly, another marked reduction appeared at 1072 cm^−1^ and correlated to C–O stretching within the cyclic ether group of GG when TA increased from 10 to 15 wt %. These observations suggest that a particular arrangement might exist in the fibers attributed to the intermolecular hydrogen bonding between TA and GG in the GG-TA matrix. From the FT-IR results, it is suggested that, after the addition of TA, new hydrogen bonds were formed between the phenolic hydroxyl of TA and hydroxyl and cyclic ether groups of the GG molecules, and thus occupied the functional group of GG matrix subsequently disrupting the existing hydrogen bonding in the GG. This was confirmed by the changes of viscosity after addition of TA to GG solutions, as shown in Table 2. These interactions are similar to the interactions in chitosan-based films for polyphenols of tea with chitosan [46]. The resultant interactions were maintained during electrospinning as H_2_O molecules evaporated, possibly in response to the alteration in fiber morphology previously shown in Figure 4.

### 3.6. XRD of GG-TA Fibrous Mats

**Figure 6:**
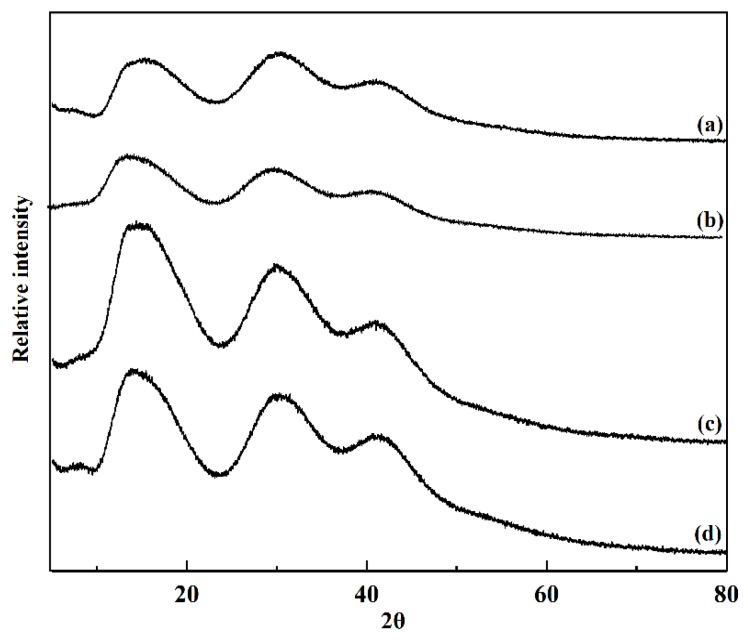
XRD study of: the neat GG (a); and of the GG fibrous mats loaded with TA with: 10% TA (b); 15% TA (c); and 20% TA (d).

The crystallinity of GG and GG-TA fibrous mats after electrospinning, as tested by XRD, is shown in Figure 6. The broad peak observed in Figure 6 indicates that neat GG exhibited low viscosity. Similar results are reported for native GG solutions [47,48]. The shape and position of the GG-TA 10% (Figure 6, Curve b), GG–TA 15% (Figure 6, Curve c), and GG–TA 20% (Figure 6, Curve d) peaks did not change when compared to the neat GG fibrous mats, which suggested that TA does not have a distinct effect on the crystallinity property of GG.

As demonstrated in the XRD profile (Figure 6, Curves a–c), the intensity of peaks increased proportionally as TA concentration increased from 10 to 15 wt %, indicating that intensity in the diffraction pattern of the GG-TA fibrous mats was strongly influenced by the presence of TA. It is worth mentioning that the diffraction peak unexpectedly decreased with higher levels of TA content (Figure 6, Curves c and d). Based on the FT-IR results, it can be assumed that hydrogen bonding between TA and GG may drive the TA molecules to gradually disperse into the GG matrix, thus interrupting the original crystal structure between GG and TA. Moreover, these two components have better miscibility at 20 wt % TA than at 15 wt % TA. A similar observation of a konjac glucomannan/curdlan blended film is previously reported with the increased addition of konjac glucomannan in the blends [49]. The XRD results show good agreement with the obtained fiber morphology, where more aggregates formed in GG-TA 15% while improved morphology was obtained in GG-TA 20%. 

### 3.7. Thermal Stability of GG-TA Fibrous Mats

**Figure 7:**
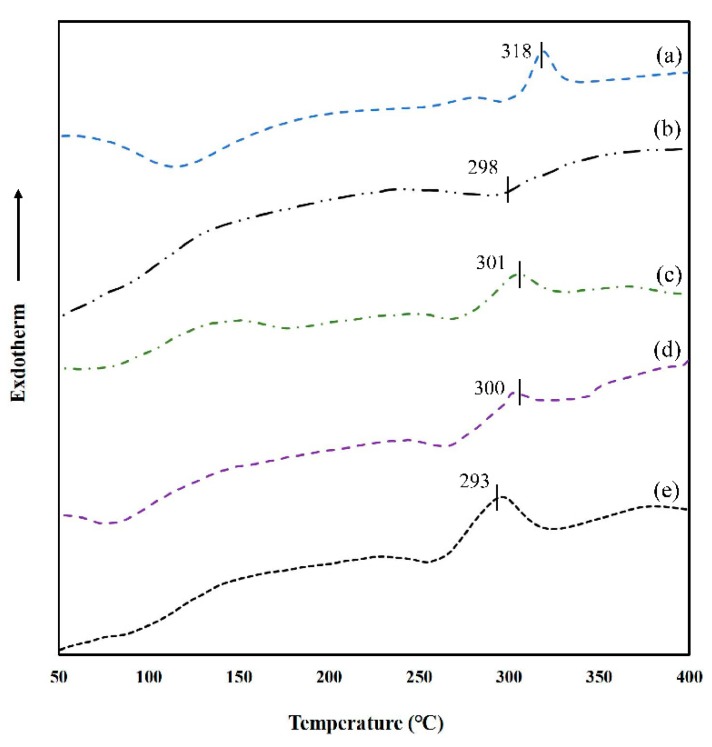
DSC thermograms of: GG powder (a); neat GG (b); and of GG fibrous mats loaded with: 10% TA (c); 15% TA (d); and 20% TA (e).

The thermal behavior of neat GG and GG-TA fibrous mats was investigated by DSC method, as depicted in Figure 7, and GG powder exhibited two endothermic peaks (278 and 318 °C), which correspond to similar thermograms that show the peaks occurring at 257.81–261.95 °C and 302.46–311.57 °C (Figure 7, Curve a), as reported by Razmkhah [50]. However, the neat GG fibrous mat exhibited only one exothermic peak (298 °C). This was attributed to the orientation effect, due to the high speed elongation and rapid solidification during electrospinning [51] (Figure 7, Curve b). All of the GG-TA fibrous mats showed a major degradation curve at around 300 °C (Figure 7, Curves a–c), which can be explained as the result of the disintegration of the polysaccharide backbone. 

Increasing the TA concentration resulted in a slight decrease to the decomposition Tm. Taking FT-IR results into account, this was attributed to the deformation of the strong H-bond network in the phenolic hydroxyl of TA, and hydroxyl and cyclic ether groups of GG molecules. In agreement with the Tm reduction, ΔHm shifted to lower values when TA increased from 10 to 15 wt % (Table 3). In addition, the apparent melting enthalpy values increased from 37.53 to 122.1 J/g when TA concentration was further raised from 15 to 20 wt %, whereas the opposite trend was found for the Tm of the network. This phenomenon suggests that the greater is the amount of TA introduced into the GG matrix, the higher is the density of the ordered junction zones formed, as a result that higher enthalpy is required to disrupt inter-chain interactions, due to the dense network formed and limited segmental mobility of polymer chains. Thermal results suggest that GG-TA fibrous mats seems to be an attractive option as an edible antioxidant film for preserving any processed food which could be produced at a temperature lower than 290 °C.

### 3.8. Antioxidant Activity of GG-TA Fibrous Mats

**Figure 8:**
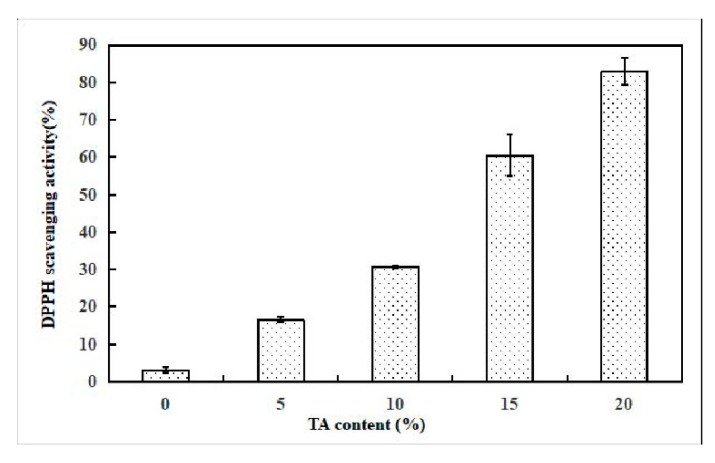
Antioxidant activity of the neat GG and of GG fibrous mats loaded with TA.

The antioxidant properties of the fibrous mats were evaluated by DPPH tests and the results, representing DPPH scavenging activity, are depicted in Figure 8. These results indicate that TA maintains inhibition activity after blending and being electrospun with GG. The DPPH scavenging activity of the TA loaded GG electrospun membranes changed from 16.6% to 80% when TA concentration increased from 5% to 20%, while the neat GG fibrous mat only exhibited a weak influence (3.5% of inhibition) on the DPPH solutions. A ~ 22 times enhancement was observed at 20 wt % TA loading in contrast with the control sample of GG without TA (*p* < 0.05), indicating a positive correlation between the content of loaded TA and the increase in the DPPH scavenging capacity.

## 4. Conclusions

This is the first report demonstrating the feasibility of using natural polysaccharide guar gum (GG) to develop electrospun GG fibrous mats with antioxidant activity without using any synthetic chemicals. The correlation between GG solution properties and the morphology of GG nanofibers was investigated prior to electrospinning. This work clearly shows that the incorporation of different ratio of TA into the GG fibrous mats leads to dramatic effects on the composite fiber morphology. Intermolecular interaction between TA and GG were confirmed to be hydrogen bonding by FT-IR, and the crystallinity and thermal stability showed varied changes accordingly. Fibrous mats loaded with TA provided excellent antioxidant activity. The characterizations presented here will aid future research in the optimization of GG-TA solution for electrospinning, potentially broadening the development of edible active packaging and delivery of therapeutics.

## Figures and Tables

**Table 1:** Incorporation efficiency of GG fibrous mats loaded with TA.

GG-TA fibrous mat	10% TA	15% TA	20% TA
Actual concentration (%)	9.98 ± 0.98 ^a^	15.6 ± 0.29 ^b^	20.01 ± 2.49 ^c^
Incorporation efficiency (%)	99.8	100.4	100.0

The data are expressed as mean ± standard derivation. Different letters (a–c) represent the data on the same row are significantly different (*p* < 0.05).

**Table 2:** Viscosity of GG and various GG-TA solutions.

Sample	GG	GG-10% TA	GG-15% TA	GG-20% TA
Viscosity (Pa· s)	204.41 ± 23.2 ^a^	270.58 ± 43.5 ^b^	290.23 ± 45.2 ^c^	312.08 ± 52.6 ^d^

The data are expressed as mean ± standard derivation. Different letters (a–d) represent the data on the same row are significantly different (*p* < 0.05).

**Table 3:** Thermodynamic properties of the neat GG and GG fibrous mats loaded with TA.

Sample	GG-powder	GG-fiber	10% TA	15% TA	20% TA
Tm (°C)	318	298	301	300	293
ΔH (J/g)	47.76	82.14	49.68	37.53	122.10
